# Scoping review of clinical, laboratory, histopathological and imaging predictors of relapse in adults with biopsy- or temporal artery ultrasound–confirmed giant cell arteritis on standard therapy with glucocorticoids alone or in combination with biologics

**DOI:** 10.1093/rap/rkag099

**Published:** 2026-08-08

**Authors:** Katarzyna Nowak, Sarah Black, Sarah L Mackie, Bogdan Kolarz, Ashley Elliott

**Affiliations:** Department of Rheumatology, Musgrave Park Hospital, Belfast, Northern Ireland; Department of Rheumatology, Musgrave Park Hospital, Belfast, Northern Ireland; Leeds Institute of Rheumatic and Musculoskeletal Medicine, University of Leeds, Leeds, UK; NIHR Leeds Biomedical Research Centre, Leeds Teaching Hospitals NHS Trust, Leeds, UK; Department of Rheumatology, Medical College, University of Rzeszow, Rzeszow, Poland; Department of Rheumatology, Musgrave Park Hospital, Belfast, Northern Ireland; Wellcome-Wolfson Institute for Experimental Medicine, Queen’s University Belfast, Belfast, Northern Ireland

**Keywords:** cranial giant cell arteritis, GCA, relapse, imaging, histopathology, clinical, laboratory

## Abstract

**Objective:**

This literature review aims to summarise current evidence on clinical, laboratory, histopathological and imaging predictors of relapse in adults with biopsy- or imaging-confirmed GCA.

**Methods:**

A comprehensive search of Ovid MEDLINE ALL and Embase databases identified 1030 articles. After screening, 58 studies met inclusion criteria. Data were extracted and thematically analysed across four domains: clinical, laboratory, imaging and histopathology characteristics.

**Results:**

Relapse occurs in >40% of patients on glucocorticoid monotherapy and ≈20% on tocilizumab, most commonly within 2 years from diagnosis. PMR symptoms and headache are the most common clinical manifestations at relapse. Laboratory markers such as ESR and CRP are widely used but lack specificity. Higher inflammatory burden at diagnosis, reflected by anaemia or thrombocytosis, correlates with future relapse. Emerging biomarkers—including calprotectin, osteopontin, IL-6, angiopoietin-2 and circulating T peripheral helper cells—show promise but require validation. Temporal artery ultrasound findings such as higher baseline halo counts, delayed ultrasound remission and limited improvement in quantitative scores are the most reliable relapse predictors. Fluorodeoxyglucose PET has limited value in predicting relapse. MRI vessel wall enhancement may correlate with relapse, but evidence remains limited to small cohorts. Histological features such as greater arterial inflammation, higher giant cell density and intraluminal thrombosis are linked to a more relapsing disease course.

**Conclusion:**

Relapse in GCA is multifactorial and cannot be predicted by a single parameter. Integrating clinical, laboratory, imaging and histological data may improve risk stratification. Prospective studies are needed to validate predictive models and enable personalised treatment approaches.

Key messages

- Relapse in giant cell arteritis is driven by complex interaction of clinical phenotype, systemic inflammation, vascular involvement, imaging abnormalities and tissue-level immune response.
- Relapse prediction cannot be adequately captured by a single clinical, laboratory, imaging or histological marker; rather, the future of personalised care will depend on the integration of these domains into validated risk-stratification models.
- The review highlights the growing role of quantitative ultrasound assessment, particularly OMERACT GCA Ultrasound Score, as a prognostic tool, with higher baseline scores associated with subsequent relapse.

## Introduction

GCA is the most common form of primary vasculitis affecting adults. Although traditionally regarded as a single disease entity, GCA encompasses at least four overlapping phenotypes: the cranial phenotype with predominant involvement of temporal arteries (cGCA); the large-vessel phenotype, which mainly affects the aorta and its major branches (LV-GCA); the PMR phenotype and the mixed phenotype. Typical presenting features of cGCA include temporal headache, jaw claudication, visual disturbance, constitutional symptoms and/or manifestations of PMR [1]. Glucocorticoids (GCs) remain the cornerstone of treatment; however, sustained long-term remission with GC monotherapy is achieved in only 10–20% of cases [2].

The introduction of GC-sparing agents has improved management strategies. Tocilizumab, an IL-6 receptor antagonist, is now widely used, and more recently the Janus kinase 1 (JAK1) inhibitor upadacitinib has demonstrated efficacy in refractory and relapsing disease [3, 4]. The treat-to-target recommendations in GCA and PMR emphasise the importance of close and regular monitoring to achieve disease control, reduce complications and minimise treatment-related adverse effects [5].

Historically, diagnosis has relied on clinical assessment, acute phase reactants such as CRP and ESR and temporal artery biopsy (TAB). In recent years, vascular US (VUS) has emerged as a key diagnostic tool. The 2023 EULAR update on recommendations for the use of imaging in LV-GCA highlights that temporal and axillary artery ultrasound (TAUS) should be considered the first-line imaging modality in patients with suspected GCA [6]. Alongside advances in VUS, interest has grown in molecular biomarkers for disease monitoring and relapse prediction [7]. Traditional inflammatory markers lack specificity, particularly when distinguishing disease activity from infection or other inflammatory conditions [5]. Emerging proteomic approaches may facilitate identification of novel biomarkers for relapse prediction [8].

Despite advances in diagnosis and treatment, relapse remains a major cause of morbidity in GCA, occurring in >40% of patients treated with GC monotherapy and ≈20% of those receiving tocilizumab. Most relapses occur within the first 2 years after diagnosis [9]. Early identification of at-risk individuals could facilitate personalised treatment strategies and reduce cumulative GC exposure and its associated complications.

## Methods

Numerous studies have examined potential predictors across clinical, laboratory, imaging and histopathological domains, but the evidence has not been comprehensively synthesised. This scoping review aimed to summarise current evidence on predictors of relapse and identify areas requiring further research.

The specific review question to be addressed is: In adults with biopsy- or TAUS-confirmed GCA on standard therapy, with GCs alone or in combination with biologics, what are the clinical, laboratory, tissue or imaging predictors of relapse?

The full study protocol has been published separately. The supplementary material (Supplementary Data S1) includes the methods and analysis sections as well as the full search strategy (Supplementary Data S2).

Studies meeting the inclusion criteria were grouped into one of the following predictors of flare categories: imaging, laboratory markers, tissue markers and clinical features. The literature search identified 1030 articles. Following screening, 58 studies were included in the final analysis. The Preferred Reporting Items for Systematic Reviews and Meta-Analyses flow diagram is provided in Supplementary Fig. S1.

The findings are presented thematically, with detailed study characteristics provided in tabular format (Table 1). Detailed study characteristics and effect estimates are provided in Supplementary Table S1.

**Table:** **Table 1** Predictors of relapse across clinical, laboratory, imaging and histological domains.

Domain	Predictor	Association with relapse	Supporting studies
Clinical	Female sex	Increased risk of relapse. Male gender is a protective factor against relapse	Alba *et al.* [9], Labarca *et al.* [13], Moreel *et al.* [16], De Miguel *et al.* [17], De Mornac *et al.* [18]
Clinical	Large vessel/aortic involvement	Baseline large vessel involvement is an independent risk factor for relapse. Baseline aortitis predicts a chronic/relapsing course	Moreel *et al.* [1], Moreel *et al.* [16], Dumont *et al.* [20], Guarda *et al.* [21], Samson *et al.* [22], Sim *et al.* [23], Espitia *et al.* [24]
Clinical	PMR features	PMR symptoms at presentation increase risk of relapse compared with those without. PMR symptoms are frequently present at relapse	Martín-Gutiérrez *et al.* [10], Muratore *et al.* [11], Restuccia *et al.* [12], Labarca *et al.* [13], Martinez-Lado *et al.* [14], Moreel et al. [15], Moreel *et al.* [16], Dumont *et al.* [20]
Clinical	Fever ≥38°C at diagnosis	Increased relapse risk	Muratore *et al.* [11], Restuccia *et al.* [12]
Clinical	Jaw claudication	Associated with relapse in biopsy proven cGCA	Sim et al. [23]
Clinical	Peripheral musculoskeletal manifestations	Increased relapse risk	De Mornac *et al.* [18]
Clinical	Venous thrombosis or leg claudication at presentation	Earlier relapse	Labarca *et al.* [13]
Clinical	Developing diabetes after diagnosis	Higher relapse compared with those with pre-existing or no diabetes	Ma *et al.* [26]
Clinical	Hypertension and diabetes at time of diagnosis	Increased relapse risk	Labarca *et al.* [13]
Clinical	Higher body weight	Increased relapse risk	Tuckwell *et al.* [19]
Clinical	Baseline depression	Increased relapse risk	Tuckwell *et al.* [19]
Clinical	Baseline osteopenia/osteoporosis	Increased relapse risk	Tuckwell *et al.* [19]
Clinical	Tocilizumab timing	Initiation >6 weeks post-diagnosis or >6 months post-diagnosis increases risk	Martín-Gutiérrez *et al.* [10], Clément *et al.* [25]
Clinical	Absence of ischaemic manifestations at diagnosis in tocilizumab treated patients	Increased relapse risk after tocilizumab initiation	Clément *et al.* [25]
Clinical	Relapse prior to tocilizumab treatment	Increased relapse risk	Clément *et al.* [25]
Clinical	Absence of tapering to <5 mg in the previous 6 months in tocilizumab-treated patients	Increased relapse risk	Clément *et al.* [25]
Clinical	Rapid steroid tapering	Increased relapse risk	Alba *et al.* [9], De Miguel *et al.* [17]
Laboratory biomarkers	Systemic Inflammation (ESR, CRP, haemoglobin)^a^	High ESR/CRP and anaemia (haemoglobin <12 g/dl) at diagnosis predict relapse	Lado *et al.* [14], Samson *et al.* [22], Martinez- Gudmundsson *et al.* [30], Visvanathan *et al.* [31]
Laboratory biomarkers	Low baseline circulating Tph cells	Circulating Tph frequency <1.0 predicted relapse	Monjo-Henry *et al.* [36]
Laboratory biomarkers	Elevated IL-6	Associated with disease relapse and activity	Weyand *et al.* [34], García-Martínez *et al.* [35],
Laboratory biomarkers	Elevated inflammatory cytokines (TNF-α, IL-12p40, PDFG, ICAM-1)	Higher in relapsing patients	Visvanathan *et al.* [31]
Laboratory biomarkers	Calprotectin, IL-23, BAFF, osteopontin, angiopoietin-2	Potential biomarkers but insufficient evidence	Tomelleri and Dejaco [2]
Laboratory biomarkers	Complement C4	Correlates with disease activity. Should be evaluated as a potential biomarker	Conticini *et al.* [32]
Laboratory biomarkers	aCL antibodies	Increase in aCL antibody levels is useful in the detection of relapses	Liozon *et al.* [33]
Imaging—ultrasound	High baseline halo count	Associated with relapse	Esperança Almeida *et al.* [37]
Imaging—ultrasound	Increase in IMT	Associated with relapse	Ponte *et al.* [44], Bosch *et al.* [46], Schäfer *et al.* [49]
Imaging—ultrasound	High baseline OGUS	Higher relapse rate	Monti *et al.* [38]
Imaging—ultrasound	Limited improvement in OGUS with treatment	Associated with higher relapse risk	Molina-Collada *et al.* [48]
Imaging—ultrasound	Neovascularisation (SMI)	Visualisation of neovascularisation via SMI may facilitate diagnosis of relapse	Skoog *et al.* [50]
Imaging—PET-CT	FDG uptake in limb arteries at diagnosis	Associated with relapse	Peyrac *et al.* [39]
Imaging—MRI	Increased vascular enhancement on vessel wall MRI	Associated with relapse	Zeng *et al.* [57]
Histology	Severe inflammatory infiltrate	Associated with relapse	Alba *et al.* [9], Restuccia *et al.* [12]
Histology	Higher frequency of giant cells	More frequent in relapsing patients	Alba *et al.* [9]
Histology	Increased CD68^+^ cells	Higher count per slice in patients with recurrence	Sultan *et al.* [59]
Histology	CCL2 (MCP-1) expression	Upregulated and more abundant in patients with multiple relapses	Cid *et al.* [61]
Histology	Weak IL-17A expression	Stronger expression linked to fewer relapses and earlier steroid discontinuation	Espígol-Frigolé *et al.* [60]

a ESR and CRP are non-specific and have limited predictive value when used in isolation.

## Results

### Clinical

At relapse, the most frequently observed clinical manifestations are PMR symptoms and/or headache [10–14]. Isolated PMR represents the most prevalent first relapse phenotype (46%; *P* < 0.001) [15, 16]. Furthermore, ≈20% of patients experience relapses characterised by new symptom categories that were not present at baseline, reflecting the heterogeneous and evolving nature of the disease.

Female sex is consistently reported as a risk factor, with women demonstrating an ≈1.43-fold higher likelihood of relapse compared with men [9, 13, 16–18]. This reflects the GCA incidence, which is two to three times more common in women [19]. Cardiometabolic conditions, including hypertension and diabetes present at diagnosis, have been linked to increased relapse frequency [13].

Large-vessel (LV) involvement, including aortitis, is among the most consistently reported predictors of relapse [16, 20]. Baseline LV involvement is associated with recurrent LV disease, greater relapse risk and a reduced likelihood of GC discontinuation, although much of the evidence is retrospective [1, 7, 21]. Jaw claudication and aortitis have also been identified as independent relapse predictors [22–24].

Treatment-related factors, which are assessed mainly during follow-up, may influence relapse outcomes, particularly in patients receiving tocilizumab. Clément *et al.* [25] described four factors increasing the risk of relapse after tocilizumab initiation: absence of ischaemic signs (defined as jaw claudication, peripheral arterial disease, superficial signs of temporal artery injury, scalp tenderness or necrosis, blindness) at the initial clinical presentation (*P* = 0.006); relapse before commencement of tocilizumab (*P* = 0.03); absence of tapering ≤5 mg of steroids for patients treated at least 6 months before inclusion (*P* = 0.03) and introduction of tocilizumab >6 months after initial diagnosis (*P* = 0.005).

While the initial GC dose has not been clearly associated with relapse risk, patients who relapse tend to accumulate higher cumulative prednisolone doses at 1 year and have longer treatment durations [9, 11]. Faster steroid tapering, particularly in the early phase, may also contribute to increased relapse rates, as observed in patients with subclinical GCA. In a study by De Miguel *et al.* [17] looking at the clinical significance of subclinical GCA in PMR patients, they observed that those patients with subclinical GCA relapsed approximately four times more frequently compared with those with isolated PMR. Those who relapsed had a faster prednisolone dose tapering in the first 3 months compared with the non-relapsed patients.

Comorbidities and medications may influence outcomes. Development of diabetes after diagnosis has been associated with reduced relapse-free survival, whereas angiotensin receptor blocker (ARB) use has been linked to longer relapse-free survival and successful GC discontinuation [26, 27]. Statins have not been associated with relapse risk [26].

### Laboratory

Laboratory biomarkers in GCA can be divided into those used to monitor disease activity and those measured at diagnosis to help predict long-term outcomes.

In this scoping review, we analysed 10 studies (listed in the supplementary material) examining potential novel blood biomarkers in GCA. At present, the detection of relapse relies heavily on traditional inflammatory markers, i.e. CRP and ESR. While widely used, these markers have important limitations, most notably their lack of sensitivity in tocilizumab-treated patients. A strong systemic inflammatory response—evidenced by clinical features such as weight loss, fever and anorexia, alongside laboratory findings including anaemia, leucocytosis, thrombocytosis and elevated ESR, CRP, fibrinogen or haptoglobin—has been associated with an increased risk of relapse [9, 12, 22]. Anaemia at diagnosis (haemoglobin <12 g/dl) has been identified as an independent predictor of relapse, further highlighting the potential prognostic value of baseline laboratory findings [14].

Fibrinogen has been explored as a potential adjunctive biomarker. Miller *et al.* [28] demonstrated that a cut-off value of 4.7 g/l provides sensitivity and specificity comparable to CRP and ESR when adjusted for corticosteroid exposure. In addition, fibrinogen may contribute to flare detection, offering greater specificity but lower sensitivity than CRP and ESR.

Despite these advances, there remains a clear need for GCA-specific biomarkers capable of identifying patients at high risk of relapse. Early studies evaluating markers of endothelial dysfunction, including von Willebrand factor and plasminogen activator inhibitor, demonstrated limited value for diagnosis, prognosis or monitoring [29].

Although plasma viscosity, ESR and CRP were associated with relapsing disease, none demonstrated sufficient specificity for relapse prediction. Plasma viscosity did not outperform ESR or CRP, despite being elevated in patients who experienced flares [30, 31].

Complement components C3 and C4 have also been evaluated as potential biomarkers. In a study involving 139 patients, including 23 who relapsed, C3 levels differed significantly between remission and relapse, whereas C4 did not. Despite this finding, neither marker proved sufficiently reliable for clinical use, as normal complement levels do not exclude active vasculitis [32].

aCL antibodies have demonstrated more promising results. In a study of 58 patients with biopsy-proven GCA, including 27 who experienced relapse, significant increases in aCL antibody levels were observed during 20 disease-related inflammatory episodes. Liozon *et al.* [33] reported sensitivity and specificity for detecting relapse were 74% and 100%, respectively, suggesting that aCL antibodies may be particularly useful in distinguishing disease flares from infection.

There are also novel biomarkers with potential roles in disease monitoring. These include serum calprotectin, IL-23, B cell activating factor (BAFF), osteopontin, angiopoietin-2 and IL-6. Some of those markers have not been discussed in detail in this review because their primary role is in disease activity assessment and monitoring rather than in predicting relapse. As the main objective of this review is to identify predictors of relapse, we focused on factors with greater relevance to relapse risk and prognostication.

Osteopontin is expressed by cells involved in both innate and adaptive immune responses. Notably, higher osteopontin levels have been associated with an increased risk of GCA relapse [2].

Angiopoietin-2 is a marker of angiogenesis. Its elevated levels were seen in patients who developed disease flares within the subsequent 4 months [2].

IL-6 is one of the most extensively studied cytokines in GCA. Its levels generally correlate with CRP, but not in patients treated with tocilizumab. Following initiation of tocilizumab therapy, IL-6 levels typically increase before gradually decreasing. Importantly, the absence of sustained suppression of IL-6 after discontinuation of tocilizumab has been associated with an increased risk of relapse [2]. IL-6 elevation has been reported in 89% of GCA recurrences compared with 58% of flares associated with elevated ESR (*P* = 0.03) [34]. Additionally, higher levels of IL-6 and TNF-α have been linked to increased relapse risk during long-term follow-up, as demonstrated by García-Martínez *et al.* [35].

Peripheral helper T (Tph) cells and follicular helper T (Tfh) cells, both CD4^+^ T cell populations, are known to be elevated in autoimmune diseases. In GCA, increased frequencies of circulating Tph and Tfh cells have been observed at diagnosis. Interestingly, lower baseline frequencies of these cells have been associated with a higher risk of relapse. A circulating Tph cell frequency <1.0 has been shown to predict relapse with high sensitivity (90%) and specificity (93%) and is associated with a significantly reduced probability of relapse-free survival [36].

While traditional inflammatory markers remain central to GCA diagnosis, numerous novel biomarkers—ranging from circulating proteins to immune cell subsets—have shown promise in improving disease assessment and relapse prediction. However, most evidence is derived from small studies, and further large-scale research is required to validate these findings and establish their role in routine clinical practice.

### Imaging

Imaging predictors were considered separately according to whether they were assessed at baseline or during follow-up, reflecting their differing prognostic and monitoring roles.

### Imaging predictors of relapse at baseline

TAUS is widely used for diagnosis, but its prognostic role is less established. Thirteen studies evaluated US predictors of relapse; however, varying relapse definitions limited direct comparison.

Higher baseline halo counts have been associated with increased relapse risk [37]. Quantitative approaches such as the OMERACT GCA Ultrasound Score (OGUS) have also shown promise as prognostic tools. Monti *et al.* [38] demonstrated that higher baseline OGUS and delayed early improvement predicted relapse; a 1-point increase in baseline OGUS increased relapse risk 2.79-fold, whereas a 10% improvement reduced risk by 33% [38]. The role of OGUS in guiding individual treatment decisions has not yet been validated.

In fluorodeoxyglucose (FDG)-PET/CT studies, Peyrac *et al.* [39] identified limb artery FDG uptake at diagnosis as an independent predictor of relapse. Patients with limb artery involvement had higher PET vascular activity scores (PETVASs) at diagnosis and significantly higher relapse rates than those without limb involvement. These findings support a potential role for FDG-PET at diagnosis as a risk stratification tool rather than a means for routine relapse monitoring.

### Imaging parameters during follow-up associated with relapse (monitoring)

The role of imaging in detecting relapse during follow-up remains less well established. In a prospective cohort study, TAUS demonstrated only moderate performance for relapse detection (sensitivity 61.2%, specificity 72.3%), supporting recommendations that US findings should be interpreted alongside clinical assessment and laboratory markers [40, 41].

Monti *et al.* [42] compared a fast-track pathway with conventional care, in which patients were assessed within one working day by an expert rheumatologist performing clinical examination and TAUS; those seen before implementation formed the conventional cohort. TAUS at relapse showed active disease in 42.9% and 45.5% of patients in the fast-track and conventional groups, respectively.

Relapses generally appear to involve less extensive arterial disease than at diagnosis. Halo resolution occurs rapidly following treatment, typically within weeks, and relapsing patients demonstrate fewer affected segments and lower intima-media thickness (IMT) burden than at disease onset [43, 44].

Conversely, Füessl *et al.* [45] found limited diagnostic utility of TAUS for relapse, with only 3.6% of patients showing ≥0.3-mm increases in superficial temporal artery IMT from prior measurements. Modest increases were more common in the facial and axillary arteries (18.4% and 21.4%, respectively). The 0.3-mm threshold, likely reflecting measurement variability and informed by prior US research, may have missed smaller but clinically relevant changes, potentially explaining the negative findings. Bosch *et al.* [46] similarly found small but statistically significant increases in axillary IMT at relapse (0.18 mm), demonstrating an association with relapse that may reflect population-level trends rather than individually actionable changes [46].

Distinguishing active vasculitis from chronic vessel wall remodelling remains a major challenge. Positive TAUS findings were observed in both asymptomatic and relapsing patients, although extensive halo burden was largely confined to new or flaring disease [47].

Longitudinal OGUS assessment may provide additional prognostic information. Improvement in OGUS was observed in non-relapsing patients but not in those who relapsed, while relapsing patients demonstrated higher mean IMT and OGUS values than at preceding assessments [48, 49].

Advanced techniques such as superb microvascular imaging (SMI) may help differentiate active from chronic disease. Skoog *et al.* [50] showed that detection of neovascularisation alongside vessel wall changes supported relapse diagnosis in a small cohort, indicating potential added value.

FDG-PET findings during follow-up have been inconsistent. Persistent uptake during clinical remission is common and may reflect vascular remodelling rather than active vasculitis. Several studies found no association between PET activity, PETVAS or total vascular score and subsequent relapse risk despite reductions in imaging scores over time [51–55]. Although one mixed-vessel vasculitis cohort reported an association between high PETVAS during remission and future relapse, this finding has not been consistently replicated in GCA-only populations [56].

Overall, FDG-PET is sensitive for detecting inflammation but has limited and variable value in predicting relapse. FDG-PET may have a role in selected diagnostic or prognostic contexts but is not supported for routine monitoring.

MRI is an emerging modality for GCA monitoring, although evidence is limited, with only two studies identified, and its utility is further constrained by limited access in clinical practice.

Zeng *et al.* [57] showed that cranial vessel wall MRI (vw-MRI) enhancement correlated with clinical relapse, with increased enhancement in relapsing patients and improvement in sustained remission. These findings suggest that increasing cranial vessel wall enhancement on vw-MRI may reflect clinically meaningful relapse, particularly in cGCA. PMR relapses showed persistent but non-progressive changes.

The GUSTO substudy (Christ *et al.* [58]) demonstrated that MRI abnormalities resolve more slowly than clinical and biochemical remission, often persisting beyond 24 weeks. Large vessel and PMR-related findings frequently remained despite clinical remission, limiting specificity.

Although promising, MRI data are based on small heterogeneous cohorts. Further prospective studies are required to determine its role and optimal timing in routine monitoring.

Overall, imaging modalities appear useful for assessing disease activity, but their role in predicting or reliably detecting relapse during follow-up remains limited. Persistent abnormalities frequently reflect chronic structural change rather than active vasculitis.

### Histopathology

With increasing use of TAUS, invasive biopsies are performed less often. However, biopsy findings remain important, as certain histological features may indicate a more persistent disease course and influence prognosis.

Moderate to severe arterial inflammation, higher numbers of giant cells and the presence of intraluminal acute thrombosis have all been associated with relapsing disease [9, 12].

Sultan *et al.* [59] investigated the role of CD68^+^ macrophages in TABs. Patients requiring immunomodulatory therapy showed higher CD68^+^ cell counts per slice compared with those who did not. Similarly, individuals who later relapsed had higher CD68^+^ counts than those who remained in remission (2.4 *vs* 1.13 cells per slice), suggesting a potential association with disease recurrence.

Significantly higher levels of IL-17A expression have been found in cGCA patients compared with controls. Interestingly, stronger IL-17A expression was linked to fewer relapses and earlier tapering or discontinuation of prednisolone [60].

Gene expression studies have identified CCL2 (MCP-1) as a potential marker of disease persistence. Higher expression levels were found in patients with confirmed cGCA, particularly in those experiencing multiple relapses, indicating an association with ongoing disease activity [61].

Predictors of relapse across clinical, laboratory, imaging and histological domains are summarised in Table 1.

## Discussion

In this scoping review, we summarised current evidence on clinical, laboratory, imaging and histopathological predictors of relapse in adults with GCA. Relapse remains common in patients receiving either GC monotherapy or tocilizumab. Although definitions of relapse and monitoring strategies vary across studies, several consistent patterns emerge from the reviewed literature.

Clinical predictors of relapse include female sex, PMR, large-vessel involvement and aortitis, while associations with comorbidities such as hypertension, diabetes, obesity and depression have been reported less consistently. Notably, 20% of patients relapse with new symptoms not present at diagnosis, underscoring the need for broad clinical vigilance. Collectively, these findings suggest that both disease phenotype and host factors contribute to relapse risk.

Predictors of relapse in tocilizumab-treated patients appear to differ somewhat from those observed in conventionally treated cohorts, although evidence remains limited and largely retrospective.

Limited evidence suggests ARBs may improve relapse-free survival, whereas statins do not significantly affect outcomes.

Traditional inflammatory markers remain important for disease monitoring but are insufficiently specific for relapse prediction. Several novel biomarkers, including aCL antibodies, serum calprotectin, osteopontin, IL-6, angiopoietin-2 and circulating Tph cells, show promise; however, evidence is largely derived from small cohorts and none are currently validated for routine clinical use.

Imaging is increasingly important in relapse assessment. Among available modalities, US currently provides the most promising prognostic data, particularly through quantitative measures such as halo counts and OGUS. However, persistent vascular wall abnormalities may reflect remodelling rather than active disease, limiting interpretation. In contrast, FDG-PET has shown inconsistent prognostic value, while MRI evidence is promising but currently limited.

Histopathological findings suggest that greater inflammatory burden within affected arteries is associated with a more relapsing disease course. Emerging molecular markers identified in biopsy tissue may improve future risk stratification, although evidence remains preliminary.

Relapse in GCA is multifactorial and cannot be predicted by any single clinical, laboratory, imaging or histological parameter. The most consistent associations identified in this review relate to large-vessel involvement, systemic inflammatory burden and quantitative US abnormalities. Future prospective multicentre studies should focus on developing validated composite prediction models integrating these domains to support personalised treatment strategies.

## Supplementary Material

rkag099_Supplementary_Data

## Data Availability

The data underlying this article are available in the article and in its online supplementary material.
