# Supplementary material for: Scoping review of clinical, laboratory, histopathological and imaging predictors of relapse in adults with biopsy- or temporal artery ultrasound–confirmed giant cell arteritis on standard therapy with glucocorticoids alone or in combination with biologics
Source: Rheumatol Adv Pract. 2026 Aug 8;10(3):rkag099. doi: 10.1093/rap/rkag099 (PMC13529366; doi:10.1093/rap/rkag099)
Supplement: Supplementary file 1 [file rkag099_supplementary_data.zip › 26-151 Supplementary Material.docx]

**Supplementary Material**

**Supplementary Table S1** – table summarising the 58 articles identified in our literature search and used for this scoping review. Included in the table are detailed study characteristics and main study findings.

| **Clinical features predicting relapse in GCA** | | | |
| --- | --- | --- | --- |
| **Author and year** | | **Study type +/- population** | **Main findings** |
| Martín-Gutiérrez et al. 2025 (10) | | *Retrospective multicentre observational study*  407 GCA patients treated with tocilizumab  63 relapsed (15.5%) | Relapse manifestations:   - PMR symptoms 47.6% - Headache 12.7%   Variables associated with relapses:   - Tocilizumab initiation > 6 weeks - Optimisation and administration of IV tocilizumab - Previous therapies to tocilizumab |
| Sim et al. 2025 (24) | | *Retrospective cohort study*  135 GCA-positive or healing/ healed arterial injury on TAB patients  25.7% relapsed in GCA-positive group  16.9% relapsed in healing/ healed arterial injury group | Features associated with relapse in GCA-positive group:   - Aortitis (p=0.007) - Jaw claudication (p=0.019)   Features associated with relapse in healing/healed arterial injury group:   - Aortitis (p=0.044) - Aortic aneurysm (p=0.047) |
| Guarda et al. 2025 (22) | | *Retrospective cohort study*  510 patients in total   - 286 biopsy-proven GCA treated without tocilizumab - 110 biopsy-negative GCA treated without tocilizumab - 114 biopsy-or imaging-proven treated with tocilizumab   303 relapsed | - 20% of patients relapsed with a symptom category absent at baseline - Majority of patients had a symptom category at first relapse that was concordant with baseline - Most common relapse features are cranial and MSK - Baseline large vessel involvement = higher risk LV involvement at relapse - 48-fold increased risk of LV features at first relapse in biopsy-proven GCA cohort with LV features at baseline not treated with tocilizumab. Not seen in tocilizumab treated group |
| Moreel et al. 2025 (1) | | *Observational retrospective cohort study*  238 GCA patients   - 169 had LVV   Of this 169 with LVV:   - 83 had PMR - 86 no PMR | - In patients without PMR, the presence of LVV is associated with relapse (p=0.011) and lower probability of stopping steroids (p=0.025) - Higher TVS at diagnosis is associated with increased risk of relapse in patients without PMR (p=0.001), but not in those with PMR symptoms |
| Alba et al. 2024 (17) | | *Review article* | - Females have 1.43 times higher risk of relapse - LVV has frequently been associated with relapsing course. In a meta-analysis the presence of LVV conferred a twofold increased risk of relapse - Presence of strong systemic inflammatory response (clinical and laboratory) is associated with increased relapse rate - Rapid decrease in steroid dose and short duration of therapy is associated with increased relapse risk - Severe inflammation and higher frequency of giant cells is seen more frequently on temporal artery biopsies of relapsing patients |
| Moreel et al. 2024 (15) | | *Retrospective cohort study*  398 GCA patients  225 relapsed | - PMR reported more frequently at the time of first relapse vs at diagnosis (60% vs 51%, p=0.027) - Isolated PMR is the most frequent disease subtype at first relapse (46%, p<0.001) - Isolated raised inflammatory markers are more frequent at the time of first relapse (11%, p<0.001) |
| De Miguel et al. 2024 (18) | | *Prospective multicentre longitudinal observational study*  150 patients   - 100 with isolated PMR - 50 with PMR and subclinical GCA   47 relapsed   - 16 in isolated PMR group - 31 in subclinical GCA group | - In isolated PMR group, only female sex was significantly associated with relapses (p=0.041) - In subclinical GCA group with a starting dose of prednisolone ≤20mg, there was 100% relapse rate - Patients with PMR and subclinical GCA had a significantly higher number of relapses (approximately 4 times more frequently) during 2 year follow up compared to isolated PMR - Patients in the subclinical GCA group who relapsed had faster prednisolone dose tapering in the first 3 months when compared to non-relapsing patients |
| Moreel et al. 2023 (16) | | *Systematic review and meta-analysis*  40 studies included | - Female sex and large vessel involvement are predictors of relapse - Females have 1.43 times higher risk of relapse (p<0.05) - Large vessel involvement is associated with a twofold increased risk of relapse (p<0.01) - GCA patients with PMR symptoms have 1.44 times higher risk of relapse (p=0.01) compared to those without |
| Clément et al. 2021 (26) | | *Retrospective analysis*  43 GCA patients treated with tocilizumab  34 relapsed | 4 factors were identified as increasing the risk of relapse after tocilizumab introduction:   - Absence of ischaemic signs (jaw claudication, peripheral arterial disease, superficial signs of temporal artery injury, scalp tenderness or necrosis, blindness) at the initial clinical presentation (p=0.006) - Relapse before tocilizumab (p=0.03) - Absence of tapering </= 5mg steroids for patients treated at least 6 months before inclusion (p=0.03) - Introduction of tocilizumab >6 months after diagnosis (p=0.005)   The presence of 2 or more of these criteria was associated with an HR for relapse of 6.3 (p=0.0006) after introduction of tocilizumab |
| De Mornac et al. 2021 (19) | | *Retrospective cohort study*  254 GCA patients  117 relapsed at least once 54 had multiple relapses | - Male gender is a relapse protective factor (p=0.04) - Peripheral musculoskeletal manifestations are a relapse risk factor (p=0.004) - Risk factors for multiple relapses include: peripheral MSK manifestations (p=0.014), negative TAB (p=0.015), large vessel involvement including upper limb ischaemia (p=0.001) and inflammation in axillary and brachial arteries on CT at diagnosis (p=0.04) |
| Ma et al. 2020 (27) | | *Multicentre retrospective review*  137 GCA patients | - Patients who developed diabetes after diagnosis has a lower relapse-free survival compared to those who had pre-existing diabetes or no diabetes (p<0.05) - Patients taking ARBs prior to diagnosis were more likely to successfully discontinue glucocorticoids without a flare in the following 3 months. (p<0.05) - Relapse rates did not differ between patients on statin therapy or not |
| Muratore et al. 2020 (11) | | *Retrospective cohort study*  87 GCA patients  37 relapsed | - PMR and worsening at imaging of LVV were the most frequently observed relapsing manifestations - The cumulative prednisolone dose at 1 year, the total cumulative prednisolone dose and the duration of prednisolone therapy were higher in patients with relapses (p=0.021, p<0.0001, and p<0.0001)   Relapsing patients had more frequently at diagnosis:   - Fever 38^o^C or more (p=0.03) - Visual manifestations (p=0.03) |
| Dumont et al. 2020 (21) | | *Retrospective observational cohort study*  326 GCA patients  171 relapsed | - Cranial symptoms were the most common relapse manifestation (68%) followed by PMR symptoms (54%) - LVV is an independent predictive risk factor of relapse (p=0.04) - Stroke is a protective factor against relapse (p=0.005) |
| Samson et al. 2018 (23) | | *Prospective multicentre interventional study*  20 GCA patients  10 relapsed | Those who relapsed more frequently had at diagnosis:   - Aortitis (p=0.048) - Or CRP>70mg/L (p=0.036) - Or haemoglobin </=10 g/dL (p=0.015) |
| Tuckwell et al. 2017 (20) | | *Randomised controlled trial*  251 GCA patients   - 119 with newly diagnosed GCA - 132 with relapsing GCA | - Relapsing patients were heavier – women 4.18kg (p=0.027), men 8.25kg (p=0.019) - Relapsers had higher baseline prevalence of depression (16% vs. 4%) - And osteopenia/osteoporosis (33% vs. 23%, p=0.002 and p=0.062 respectively) |
| Restuccia et al. 2016 (12) | | *Retrospective observational cohort study*  157 GCA patients  57 relapsed | - PMR (46.5%) and cranial symptoms (41.9%) were the most frequent manifestations at the time of the first relapse - In the multivariate model, fever of 38^o^C or more (p=0.03) and the severity of inflammatory infiltrate (p=0.006) were significantly associated with an increased risk of flares |
| Labarca et al. 2016 (13) | | *Retrospective cohort study*  286 GCA patients  213 relapsed | - The most experienced symptoms at the time of relapse are PMR (33.1%), headache (32.3%), malaise/fatigue (20.6%) and scalp tenderness (7.8%) - History of venous thrombosis (p=0.024) and presentation with leg claudication (p=0.043) are associated with earlier relapse - More patients with established hypertension (p=0.007) and diabetes (p=0.039) at diagnosis were in the high relapse rate group and more females were in the low or high relapse groups than in the no relapse group (p=0.034) |
| Alba et al. 2014 (28) | | *Longitudinal cohort study*  106 GCA patients | - Patients receiving ARB had significantly longer relapse-free survival than patients treated with ACEI or patients not receiving ACEI/ARB (p=0.02) - There were no frequent relapsers (patients who relapsed 3 times or more) among patients receiving ARB compared with 6% among those treated with ACEI and 21% among those with no ACEI/ARB - Patients who received ARB achieved a prednisolone maintenance dose <10mg/day faster than all other patients (p=0.0002) |
| Espitia et al. 2012 (25) | | *Retrospective cohort study*  22 biopsy-proven GCA patients  10 patients had aortitis on CT | - 6 patients in GCA-Ao group had at least one relapse as compared to 3 patients in the other group (p=0.14) - 5 patients in GCA-Ao group had multiple relapses, whereas none of the patients in the GCA-noAo group had multiple relapses (p=0.012) - Mean number of relapses per patient were 1.6 in GCA-Ao group and 0.25 in GCA-noAo group (p=0.037) - Aortic involvement at onset could predict a more chronic/ relapsing course |
| Martinez-Lado et al. 2011 (14) | | *Population based observational cohort study*  174 biopsy-proven GCA patients  71 relapsed | - Headache (52%) is the most common feature at the time of relapse - PMR occurs in 30% of patients - The presence of anaemia (Hb<12 g/dL) at the time of disease diagnosis is the best predictor of relapses or recurrences of GCA (p=0.04) |
| **Biomarkers predicting relapse in GCA** | | | |
| **Author and year** | | **Study type +/- population** | **Main findings** |
| Kudraszew et al. 2025(35) | | *Review article* | - Significant difference between serum CLP levels in patients with active versus inactive disease - CLP levels did not predict relapse |
| Monjo-Henry et al., 2025 (38) | | *Prospective observational study*  65 newly diagnosed GCA patients  46 completed follow up  19 patients relapsed | - A cTph cell frequency <1.0 predicted relapse with a sensitivity of 90% and specificity of 93% - A significant difference in the probability of relapse-free follow-up was observed between the group of patients with baseline cTph cell frequency ≥1.0 and the group with a basline cTph cell frequency <1.0 (p<0.0001) |
| Tomelleri et al. 2024 (2) | | *Review article* | - 3 studies found positive correlation between calprotectin levels and disease activity of GCA. 1 study did not confirm this result - Evidence for the value of IL-23 as a biomarker in GCA is limited - B cell activating factor (BAFF) levels are higher in patients with new-onset GCA than in healthy controls, and serum concentrations strongly correlate with disease activity - Correlation with disease activity has also been reported for osteopontin and relapses might be predicted by baseline levels of osteopontin (higher levels appear to be linked to increase relapse risk) - IL-6 levels reflect disease activity except in patients treated with tocilizumab. However, lack of IL-6 decline is associated with higher risk of relapse once tocilizumab is stopped - None of the above markers are supported by enough evidence to qualify as routine parameter – further studies are required |
| Conticini et al. 2023 (33) | | *Retrospective observational study*  139 patients with LVV   - 28 c-GCA - 61 LV-GCA - 50 c- plus LV-GCA   23 relapsed | - C3 and C4 do not seem useful in the diagnosis of GCA, as normal values do not rule out active vasculitis - C3 and C4 correlate with disease activity with decreased levels in patients attaining remission and sustained remission - Even stronger decreases in C3 and C4 levels were noted in tocilizumab treated patients - C4 should be evaluated as potential biomarker of disease activity and treatment response |
| Visvanathan et al. 2011 (32) | | *Prospective longitudinal study*  44 newly diagnosed GCA patients  13 relapsed | - Higher ESR, CRP intercellular adhesion molecule (ICAM)-1, TNFα, IL-6, TGF-β, MMP-9, PDGF and IL-12p40 were observed in relapsing patients vs those who achieved sustained remission - Differences were statistically significant for ESR, CRP, ICAM-1 and PDGF - In patients who relapsed, significantly (<0.01) elevated median values were observed at the protocol-specified visit closest to relapse vs baseline for ESR, CRP, ICAM-1, TNFα and IL-12p40 |
| García-Martínez et al. 2010 (37) | | *Prospective cross-sectional evaluation*  54 biopsy-proven GCA patients  15 relapsed | - Patients who had experienced at least one relapse during follow-up had higher levels of TNFα and IL-6 than patients with no relapsing disease (p=0.042 and p=0.04 respectively) |
| Weyand et al. 2000 (36) | | *Prospective observational cohort study*  25 biopsy-proven GCA patients  15 relapsed | - Elevation of ESR was seen during only 58% of all disease flares, but 89% of disease recurrences were associated with increased plasma IL-6 levels (p=0.03) - Plasma IL-6 is more sensitive than ESR for indicating disease activity |
| Liozon et al. 2000 (34) | | *Prospective observational longitudinal cohort study*  58 biopsy-proven GCA patients  21 relapsed | - Significant rise in aCL was recorded in 20/27 analysable inflammatory episodes related to GCA, occurring in 21 patients - No aCL positivity was recorded in any of the 28 analysable GCA-unrelated inflammatory flares (p<0.0000001) - Serum aCL levels are useful in the detection of flares and relapses in giant cell arteritis, with 74% sensitivity and 100% specificity and can be of value in distinguishing subclinical flares from infection |
| Gudmundsson et al. 1993 (31) | | *Prospective observational cohort study*  31 GCA patients receiving prednisolone  10 relapsed | - Significantly higher values for plasma viscosity and ESR but not for CRP were found in patients who flared - The logistic regression analysis showed no overall preference between plasma viscosity, ESR, or both in predicting flare ups |
| Nordborg et al. 1991 (30) | | *Prospective observational longitudinal cohort study*  63 GCA patients  27 relapsed | - Determination of von Willebrand factor antigen and plasminogen activator inhibitor activity is of limited clinical value in the diagnosis, prognosis, and monitoring of steroid treatment in patients with GCA |
| **Imaging (ultrasound, PET-CT, MRI) predicting relapse in GCA** | | | |
| **Author and Year** | | **Study type +/- Population** | **Main findings** |
| **Ultrasound** | | | |
| Haaversen et al. 2024 (42) | | *Prospective observational cohort study*  132 GCA patients  80 relapsed | - Ultrasound yielded a sensitivity of 61.2% and specificity of 72.3% for diagnosing relapse - Ultrasound demonstrated moderate sensitivity and specificity as a monitoring tool in the follow-up of GCA patients |
| Coath et al. 2021 (43) | | *Review article* | - The authors use a modified criteria for diagnosing active Takayasu arteritis to diagnose relapsing GCA:   2 of the 4 criteria helps diagnose relapsing GCA:   - Constitutional symptoms - Claudication symptoms - CRP >10 mg/l - Ultrasonographic changes of GCA |
| Monti et al. 2020 (44) | | *Retrospective observational cohort study*  160 GCA patients   - 63 evaluated via fast-track approach - 97 via conventional approach   82 patients relapsed | - Median time to disease reoccurrence of 8 months - The cumulative incidence of relapse and time to first relapse did not change after fast-track approach implementation - 14 relapsing patients in the fast-track group had TAUS. 42.9% had US signs of active disease - 22 TAUS were performed in the conventional group with 45.5% showing halos |
| De Miguel et al. 2012 (45) | | *Prospective observational longitudinal cohort study*  30 GCA patients  38 occurrences of GCA within this cohort  13 relapses | - Halo disappearance occurred in 94.7% of cases during follow up, with a mean time of around 11 weeks - The mean time until halo disappearance after initiation of corticosteroids was around 9 weeks for relapsed patients - Relapsed patients had a mean of around 2.5 branches presenting with halo - Overall, the relapse cases appeared to have less arterial wall affectation than the primary GCA cases and earlier loss of halo sign |
| Esperança Almeida et al. 2023 (39) | | *Retrospective analysis*  72 GCA patients  37 relapsed | - Patients with higher halo count were more likely to relapse. Halo count was defined as the number of vascular territories per patient showing a clear halo sign - Ischaemic features at presentation did not significantly predict relapse, nor did constitutional symptoms, cardiovascular morbidity, platelet count or pre-treatment CRP |
| Ponte et al. 2021 (46) | | *Prospective analysis*  49 GCA patients  19 patients had 32 relapses  17 patients had TAUS | - Halo sign was present in 94% of first disease relapses but with a lower mean number of segments with halo and sum of halo IMT compared with disease onset - In all 16 cases, an increase in halo IMT, with or without an increase in the number of arterial segments with halo, was observed in comparison to the previous ultrasound assessment recorded |
| Füessl et al. 2025 (47) | | *Retrospective analysis*  100 GCA patients  36 relapsed | - Patients who relapsed were significantly more likely to have thrombocytosis and anaemia at the time of diagnosis - Repeated ultrasound compared to previous result showed increase in maximum IMT (at least +0.3mm) in 3.6% of the superficial temporal arteries, 18.4% of the facial arteries, and 21.4% of the axillary arteries in patients with relapsing disease |
| Bosch et al. 2022 (48) | | *Retrospective observational cohort study*  101 GCA patients   - 67 with axillary involvement - 34 without axillary involvement | - Time until first relapse and relapse rates were similar in both groups - Relapses resulted in an increase of the IMT by 0.18mm |
| Monti et al. 2018 (49) | | *Prospective observational cohort study*  293 patients   - 118 with clinically confirmed GCA   78 assessed for possible relapse | - 24.4% of patients assessed for possible relapse had positive TAUS - Over time the number of halos reduced. Only new or flaring patients showed a halo in four or more sites - Analysis of temporal artery halo size showed even though there were no significant differences in the average size over time, halo sizes comparable with disease onset were recorded during flares |
| Molina-Collada et al. 2025 (50) | | *Retrospective observational study*  76 GCA patients  19 relapsed | - OGUS significantly improved between baseline and 6 months and from 3-6 months in non-relapsing patients - No significant changes at 3 and 6 months were observed in those who relapsed - The mean 0-6 months OGUS improvement was lower in patients who relapsed |
| Schäfer et al. 2025 (51) | | *Prospective observational cohort study*  36 GCA patients  4 relapsed | - At relapse, mean IMT and OGUS were higher when compared to preceding assessment |
| Monti et al. 2025 (40) | | *Multicentre prospective study*  97 GCA patients  35 patients experienced 1 or more relapse | - Baseline OGUS was directly associated with the risk of relapse with a significantly higher OGUS in patients experiencing a relapse - The rapidity and degree of reduction of OGUS during the first 3-6 weeks after diagnosis was associated with the risk of future relapse - 1-point increase in the baseline OGUS confers 2.79-fold increase in the risk of relapse during follow-up - 10% improvement in OGUS at any time point compared to baseline was associated with a 33% reduction in the risk of relapse |
| Skoog et al. 2024 (52) | | *Retrospective observational study*  33 GCA patients  3 relapsed | - 1 relapsed patient displayed neovascularisation together with low echogenic vessel wall swelling strongly suggesting inflammatory activity - Further studies are needed to evaluate whether SMI can provide prognostic information and to determine its role in monitoring disease activity |
| **PET-CT** | | | |
| Quinn et al. 2025 (53) | | *Prospective observational cohort study*  36 patients with GCA on tocilizumab   - 31 patients in clinical remission - 5 with active clinical symptoms   12 patients with GCA treated with glucocorticoid monotherapy | - 100% of symptomatic patients on tocilizumab had positive FDG-PET - 51.6% of patients in clinical remission on tocilizumab had active FDG-PET - No association with the presence of ongoing FDG-PET activity, or degree of FDG-PET activity, and future relapse risk was observed - PETVAS was significantly lower in the tocilizumab treated group - Once a patient discontinued tocilizumab, a subsequent increase in FDG-PET activity while off treatment preceded clinical relapse in 3 patients |
| Hemmig et al. 2024 (54) | | *Observational cohort study*  40 patients with LV-GCA discontinuing treatment  11 relapsed | - Patients who relapsed were comparable to those who remained in remission with respect to the presence of active vasculitis on MRI +/- PET-CT, the number of segments with vasculitic findings on MRI or the highest SUV artery/liver ratio on PET-CT - PET-CT or MRI at treatment cessation did not predict relapse |
| Billet et al. 2024 (55) | | *Retrospective observational study*  55 patients with LV-GCA  18 patients experienced at least one relapse | - PET scan performed at diagnosis (PET1) and during follow up (PET2) between 3-12 months after diagnosis while GCA in remission - PETVAS and TVS decreased between PET1 and PET2 in all patients (p<0.001) - Neither the PETVAS nor TVS was predictive of occurrence of subsequent relapse |
| Sammel et al. 2020 (56) | | *Prospective observational cohort study*  21 GCA patients  15 patients completed 6-month PET-CT  12 relapsed | - 57% of patients had a TVS ≥ 10 on the baseline PET/CT - 58% patients with a TVS ≥ 10 relapsed compared with 56% with a TVS < 10 - However, patients with a high TVS were numerically more likely to experience ischemic relapses (33% vs 11% *P* = .34) - For the 15 patients who had scans performed at both baseline and 6 months, the median TVS reduced from 14 to 5 (*P* < .01). This reduction was seen for both large vessel (5 to 2, *P* < .01) and cranial vessel scores (3 to 1, *P* = .01) - None of the 5 patients who had ongoing FDG vascular activity on the 6 month PET-CT scan experienced a subsequent clinical relapse |
| Peyrac et al. 2024 (41) | | *Retrospective observational study*  108 GCA patients  40 relapsed | - Relapse occurred at a median time of 14 months after diagnosis - Univariable analysis showed that only FDG uptake in limb arteries at diagnosis was associated with relapse during follow up - Multivariable Cox regression model confirmed that FDG uptake in limb arteries at diagnosis was a factor associated with GCA relapse - PETVAS at diagnosis was higher in patients with limb artery involvement compared to those without - GCA relapse occurred in 58.3% (*n* = 14/24) of patients who had FDG uptake in limb arteries at diagnosis versus 28.4% (*n* = 21/74) of patients who had no FDG uptake in limb arteries |
| Blockmans et al. 2006 (57) | | *Prospective cohort study*  35 GCA patients   - 22 patients had 2^nd^ PET at 3 months - 8 of these patients had 3^rd^ PET at 6 months   18 relapsed | - TVS fell substantially with treatment at 3 months - There was no significant difference between the TVS of the relapsing and sustained remission groups at diagnosis, 3 and 6 months |
| Grayson et al. 2018 (58) | | *Prospective cohort study*  115 patients  56 with LVV:   - 30 GCA - 26 Takayasu’s arteritis   59 disease comparators:   - 35 with hyperlipidaemia - 17 LVV mimics - 7 healthy controls | - FDG-PET performed poorly as a marker of disease activity in LVV with 57.7 patients in clinical remission had PET scan findings interpreted as ongoing vascular inflammation - 8 out of a cohort of 39 patients who underwent PET-CT during a period of clinical remission and had at least 3 months of follow up experienced a relapse. The median follow up for this cohort was 15 months - Patients were categorised during clinical remission as having a high amount of arterial FDG uptake (PETVAS ≥ 20) or a lower amount of arterial FDG uptake (PETVAS < 20) - Significantly more patients with PETVAS ≥ 20 experienced future clinical relapse compared to patients with PETVAS < 20 (45% vs 11%, p=0.03) |
| **MRI** | | | |
| Zeng et al. 2025 (59) | | Observational longitudinal cohort study  14 GCA patients  6 patients relapsed:   - 4 with cranial or ocular manifestations - 2 with PMR only   2 further patients relapsed after completion of study. | - All 4 patients who experienced cranial or ocular relapse had increasing MRI enhancement in at least one cranial structure on vw-MRI when compared to previous imaging - Cranial structures that showed increased enhancement at relapse included the occipital artery, optic nerve sheath, and maxillary artery - 2 patients who experienced relapse of PMR only had persistent but no increase in enhancement in any cranial structures - 2 further patients experienced a GCA relapse after completion of the study. One of these patients had persistent MRI enhancement at month 6 and another had an increase in MRI enhancement between months 6 and 12 - The other 6 non-relapsing patients had no increased enhancement form baseline |
| Christ et al. 2025 (60) | | *Prospective observational cohort study*  18 GCA patients  13 patients completed the study  All 13 patients were in clinical remission by week 24 | - MRIs performed at baseline, weeks 24, 52 and 104 - At week 24 inflammatory signals were still detectable in 1 in 4 cranial segments. The percentage of patients in MRI remission increased from 28% at baseline to 45% - At weeks 52 and 104, no cranial vascular segment showed active vasculitis - Large vessels except the ascending aorta showed little or no decrease in vasculitis findings - PMR imaging findings persisted in most patients with 36% of regions showing inflammatory findings at baseline compared to 19% of regions at week 104. No correlation with PMR symptoms was observed |
| **Histology features predicting relapse in GCA** | | | |
| **Author and year** | **Study type +/- Population** | | **Main findings** |
| Sultan et al. 2018 (61) | *Observational cohort study*  42 GCA patients  17 relapsed | | - Patients who developed at least one episode of symptom recurrence during follow-up period were found to have an increased number of CD68+ cells per slice in comparison to those who did not have a recurrence (2.4 cells/slice vs 1.13 cells/slice respectively, p=0.031) |
| Espígol-Frigolé et al. 2013 (62) | *Observational cohort study*  57 GCA patients | | - IL-17A expression was significantly increased in temporal artery samples from GCA patients compared with controls (p=0.016) - IL-17A mRNA concentrations in lesions tended to be higher in patients who achieved sustained remission or experienced just one disease flare compared with those who experienced multiple relapses (p=0.058) |
| Cid et al. 2006 (63) | *Observational study*  12 GCA patients   - 6 with relapsing GCA - 6 who easily achieved sustained remission | | - CCL2 (MCP-1) was up-regulated in temporal artery samples from relapsing individuals - CCL2 mRNA concentration in lesions was significantly higher than in controls (p=0.0001) - CCL2 was more abundant in patients who experienced two or more relapses during first year compared to those who endured sustained remission (p=0.0233) |

**Supplementary Figure S1** – PRISMA diagram

**Supplementary Data S1** – Methods and analysis sections of the scoping review protocol

**Protocol Design:** This protocol is reported in accordance with the PRISMA -P (Preferred Reporting items for Systematic Review and Meta Analysis Protocols) guidelines. The scoping review will be conducted in line with the JBi methodological framework for scoping reviews.

**Types of participants:** This review will include studies that involve adults (≥50 years old) of any gender with biopsy or temporal artery ultrasound confirmed GCA. There will be no geographical restrictions. Only studies published in English, and full text articles will be included.

**Types of interventions:** Eligible studies will include patients receiving standard treatment for GCA with:

- Glucocorticoids alone, or
- Glucocorticoids in combination with biologics or targeted synthetic therapies.

**Types of outcomes:** The primary outcome of interest is rates of confirmed relapse on treatment. Relapse is defined as new or recurrent disease activity after achieving remission. We will review clinical, laboratory biomarkers (including acute phase reactants, proteomic profiling, and blood-based biomarkers), tissue biomarkers and imaging findings identified as potential predictors of relapse.

**Types of studies:** The review will consider randomised controlled trials (RCT); prospective/retrospective studies, registry studies, and systematic or narrative review. Of note, studies including patients with concomitant large-vessel involvement were eligible provided the underlying population had cranial GCA.

**Criteria for excluding studies in the review:** Case reports, studies not written in English, and studies for which we were unable to access a full text article were excluded from the review.

**Search methods:** The search strategy: Electronic searches were conducted in MEDLINE(R) ALL and EMBASE. The full search strategies are presented in Supplementary material (S6).

1. Manual review of the title, study type and abstract were conducted on all identified abstracts.
2. Duplicates and studies not meeting inclusion criteria, study protocols as well as abstracts for which we are unable to obtain the full text article were removed from analysis.
3. Two reviewers independently selected abstracts against the inclusion and exclusion criteria.
4. Discrepancies in selections were resolved at a meeting between the two reviewers and an independent lead for the project.

**Critical appraisal and data collection:** Data will be extracted using a pre-defined data charting form and extracted information will include study characteristics, population characteristics, diagnostic criteria for GCA, treatment exposure, definition of relapse, type and timing of predictor assessed and key findings relevant to flare prediction. Data charting will be performed independently by the two reviewers. Consistent with JBi guidance for scoping reviews formal risk of bias will not be mandatory.

**Supplementary Data S2 – Ovid MEDLINE(R) ALL search strategy 23.09.2025 and EMBASE search strategy 26.09.2025**

Ovid MEDLINE(R) ALL <1946 to September 23, 2025>

1     giant cell arteritis.mp. or Giant Cell Arteritis/     9885

2     gca.mp.     5487

3     temporal arteritis.mp.  2421

4     Ultrasonography/  210721

5     Biopsy/     193652

6     (ultraso* or biops* or imag*).mp.   3942990

7     relaps*.mp. 251838

8     recurrence/ or symptom flare up/    209528

9     recur*.mp.  969200

10    flar*.mp.   24592

11    exacerbat*.mp.    182218

12    exp Glucocorticoids/ or glucocorticoid*.mp.     264516

13    exp Prednisolone/ or prednisolone.mp.     71967

14    exp Steroids/ or steroid*.mp. 1205493

15    exp Biological Products/ or biologic*.mp. 2652607

16    tocilizumab.mp.   7985

17    janus kinase inhibitor*.mp. or Janus Kinase Inhibitors/     4619

18    jak-i.mp.   292

19    upadacitinib.mp.  1636

20    (or/1-3) and (or/4-6) and (or/7-11) and (or/12-19)    312

21    limit 20 to english language  277

Embase <1974 to 2025 September 24>

1     giant cell arteritis.mp. or giant cell arteritis/     13510

2     gca.mp.     9414

3     temporal arteritis.mp. or temporal arteritis/   5109

4     ultrasound/ 268255

5     biopsy/     183920

6     imaging/    196853

7     (ultraso* or biops* or imag*).mp.   5173654

8     relapse/ or relaps*.mp. 517099

9     recurrent disease/      251136

10    disease exacerbation/   224798

11    (recur* or flar* or exacerbat*).mp. 1972964

12    exp glucocorticoid/ or glucocorticoid*.mp.      1006082

13    prednisolone.mp. or prednisolone/   188448

14    exp steroid/ or steroid*.mp.  2192355

15    exp biological product/ or biologic*.mp.  3293490

16    tocilizumab.mp. or tocilizumab/     35127

17    Janus kinase inhibitor/ or janus kinase inhibitor*.mp.      13096

18    jak-i.mp.   80

19    upadacitinib.mp. or upadacitinib/   5282

20    (or/1-3) and (or/4-7) and (or/8-11) and (or/12-19)    1177

21    limit 20 to english language  1137

22    conference abstract/ or conference abstract.pt. 5616957

23    21 not 22   649
